# An Insight Into the Acceptance and Hesitancy of COVID-19 Vaccines in Pakistan: A Cross-Sectional Survey

**DOI:** 10.7759/cureus.32363

**Published:** 2022-12-09

**Authors:** Arsalan Rasheed, Wajeeha Idrees, Qaisar Ali Khan, Hassan Mumtaz, Tamara Tango, Marium Aisha Mangrio, Hoor Ul Ain, Priyadharshini Saravanan, Bhavana Vattikuti, Leyla Kedir Bereka, Christopher S Farkouh

**Affiliations:** 1 Molecular Biology and Genetics, Abdul Wali Khan University Mardan, Mardan, PAK; 2 Management Sciences, University of Faisalabad, Faisalabad, PAK; 3 Internal Medicine, District Headquarter (DHQ) and Teaching Hospital Kohat Development Authority (KDA) Kohat, Kohat, PAK; 4 Urology, Guy's and St Thomas' Hospital, London, GBR; 5 General Practice, Surrey Docks Health Center, London, GBR; 6 Public Health, Health Services Academy, Islamabad, PAK; 7 Clinical Research Center, Maroof International Hospital, Islamabad, PAK; 8 Internal Medicine, Faculty of Medicine Universitas Indonesia, Jakarta, IDN; 9 Medicine and Surgery, Civil Hospital, Sukkur, PAK; 10 Internal Medicine, Jinnah Medical College, Peshawar, PAK; 11 Internal Medicine, Pondicherry Institute of Medical Sciences, Pondicherry, IND; 12 Internal Medicine, Cebu Doctors University College of Medicine, Philippines, PHL; 13 Internal Medicine, Fatima Jinnah Medical College, Lahore, PAK; 14 Dermatology, Rush Medical College, Chicago, USA

**Keywords:** survey, hesitancy, acceptance, pakistan, covid-19 vaccines

## Abstract

Background: COVID-19 vaccines are found to be effective interventions to tackle COVID-19. However, the hesitancy towards its acceptance has been rising in Pakistan. This study highlights the opinion of the general population in Pakistan regarding the acceptance and hesitancy of COVID-19 vaccination.

Methods: A descriptive cross-sectional survey study was conducted among Pakistanis from December 2021 to January 2022. Adult respondents that have and have not received COVID-19 vaccinations were included in this study. Data collection was obtained through questionnaires that assessed acceptance and hesitancy toward COVID-19 vaccines. Statistical analysis was performed using IBM SPSS software version 25 for Windows.

Results: We obtained 367 respondents with 333 respondents completing the questionnaire. There were 259 respondents who have been vaccinated. A total of 67.9% of responses agreed that vaccines could control the COVID-19 pandemic. The reasons for not getting vaccination were afraid of adverse effects (48.6%) and COVID-19 vaccines not being tested thoroughly (30.9%). The main reason for vaccine acceptance was awareness about vaccines (23.1%), a belief that vaccines can stop severe COVID-19 disease (16.8%), and self-protection (14.7%).

Conclusion: Most Pakistanis agreed that vaccines could manage the pandemic. Vaccine acceptance was contributed by the awareness and belief regarding the protective effects of vaccines while vaccine hesitancy was due to the public’s doubt about the vaccines’ side effects and testing. The Pakistan government should focus on emphasizing knowledge about vaccines, educating the vaccines’ adverse effects, and utilizing social media in doing so.

## Introduction

COVID-19 was discovered in Wuhan, China, in December 2019 [1]. COVID-19 has had a great impact on developing countries surrounding China, including Pakistan, India, Iran, Afghanistan, and Bangladesh [2]. Effective healthcare strategies are needed to tackle COVID-19. Developed countries have already defined healthcare systems and public education programs while developing countries experienced inadequate healthcare systems and insufficient education programs. Strengthening the healthcare system and its policy plays a crucial role in improving the quality of life and providing patients safety in low or middle-income countries, i.e., Pakistan [3]. The prevalence of COVID-19 remained high throughout the COVID-19 pandemic, it mainly hit the Punjab province and it was assumed to be due to overcrowding and low to medium-level economy. On 26 February 2020, the first two cases were reported in Pakistan [4,5]. While writing this article there were 973,284 confirmed COVID-19 cases and 22,582 deaths across the country [5].

After the declaration of the pandemic by WHO in March 2020, scientists and pharmaceutical companies are racing against time to develop vaccines [6]. Each year for saving millions of lives from communicable diseases, vaccination remains one of the most reliable and highly cost-effective public health interventions. [1,7]. The development of COVID-19 vaccines was aided by the knowledge about coronavirus causing SARS and MERS. By December 22, 2020 different vaccines against COVID-19 were in different stages of development, 85 vaccines were in the preclinical stage and 63 were in the clinical stage of which six were approved for early or limited use, two were approved for full use and one vaccine was abandoned. Pfizer-BioNTech’s (BNT162b2) and Moderna (mRNA-1273) mRNA vaccines have been approved for emergency use in the United States (US) [8].

Vaccines are widely recognized by health authorities and the medical community as a major tool for achieving public health successes, such as the eradication of smallpox [9,10]. Yet, for many individuals, this is not a sufficient basis to embrace vaccination wholeheartedly. According to the SAGE Working Group on Vaccine Hesitancy, vaccine hesitancy is a delay in vaccine acceptance or vaccine refusal although the vaccination services are available [11].

In Pakistan, hesitancy against vaccination still exists and it is obvious from the facts of hesitancy against routine childhood vaccination like polio, hepatitis, and measles vaccination. Different factors like social, economic and religious factors contributed to these trends [12]. On February 2, 2021, the National Institute of Health (NIH), Ministry of Health, National Command and Operation Centre (NCOC), and World Health Organization (WHO), jointly launched an Outreach COVID-19 Vaccination campaign for elderly people in Islamabad (Pakistan) [13]. During the early phase COVID-19 vaccination campaign, healthcare providers were prioritized to receive this vaccination [14]. Unfortunately, even after the vaccination program started in February 2021, only 16.7% of Pakistanis were fully vaccinated until October 2021 [15]. Until December 2022, there are three types of vaccines that have been approved and delivered to Pakistanis, including RNA vaccines (Spikevax, Comirnaty), non-replicating viral vector vaccines (Convidecia, Sputnik V, Vaxzevria), and inactivated vaccines (Covilo, Coronavax) [16]. 

The increasing prevalence rate of COVID-19 created fear among Pakistanis [17]. Considering the high prevalence of COVID-19 and the low COVID-19 coverage rate [15] in Pakistan, it is important to document and understand the level of vaccine acceptance and hesitancy in Pakistan and the reasons behind it. Therefore, this study aims to analyze the factors contributing to hesitancy and acceptance of COVID-19 vaccines among Pakistanis.

## Materials and methods

Study design

A descriptive, cross-sectional survey-based study was conducted from December 2021 to January 2022 to analyze the attitude of the general Pakistani population toward COVID-19 vaccines.

Sample size

The sample size was estimated by a sample size calculator by the Raosoft formula [18] by setting the margin of error at 5%, the confidence level at 94%, and the response distribution at 50%. Knowing that the population of Pakistan is 231,630,496 [19], we obtained the recommended minimum sample size of 354 respondents.

Data collection and management

A structured questionnaire was established in a Google Form after reviewing the relevant literature search on COVID-19 vaccination. The questionnaire was written in English, Urdu and other local languages of Pakistan. The questionnaire consisted of four sections, i.e., informed consent for the participants, demographic information, the attitude of study participants toward COVID-19 (reasons to accept), and the attitude of study participants toward COVID-19 (reasons to hesitate). The questions were close-ended to ensure the inclusion of important points and the ease of responding to the question.

Different social media platforms such as WhatsApp, Facebook, Instagram, and LinkedIn were used for the collection of data and the participants participated through google link. Convenience samplings were used in this research. The participation of the respondents was voluntary. It took around five minutes to complete the form. The privacy of the participants' data was maintained by giving access to the repository data only to core members.

Inclusion and exclusion criteria

Both vaccinated and unvaccinated adult males and females above the age of 18 years from Pakistan were included in this study. People other than Pakistan nationality, or Pakistani nationals residing outside Pakistan were excluded from the study. All participants responded through the same questionnaire.

Data analysis 

All the data in the google form was transferred to a Microsoft Excel sheet and was analyzed by IBM SPSS software version 25 for Windows.

Ethical considerations

Written informed consent was obtained from respondents before the collection of data. Ethical approval was taken from Jinnah Medical College Peshawar Pakistan Ethical Board with reference no DIR/JMCP/EB/0001.

## Results

We obtained 367 respondents with only 333 of them filling out the questionnaire completely. The respondents consisted of 77.8% males (259 out of 333) and 22.2% females (Table 1). Among the respondents, 259 of them have been vaccinated. We further divided the participants into two age groups: a group with an age range between 18 and 25 years (32.1%) and a group whose age range is between 26 and 55 years (67.9%).

**Table 1:** Age and gender distribution of the participants

Socio-demography	Frequency (n)	Percentage (%)
Gender		
Male	259	77.8
Female	74	22.2
Age		
18 - 25 years old	107	20.0
26 - 55 years old	226	80.0

A total of 67.9% of respondents agreed that the vaccines can control the COVID-19 pandemic while the remaining thought otherwise (Table 2).

**Table 2:** Question whether vaccines can control the pandemic

Question	Frequency	Percentage (%)
Do you think vaccines can control pandemic?	Yes = 226	67.9
	Maybe = 107	32.1

Regarding the main reasons for not getting vaccinated (Table 3), 48.6% of respondents were afraid of adverse effects and 30.9% of respondents agreed that COVID-19 vaccines have not been tested thoroughly. The hesitancy toward COVID-19 vaccination was also due to mistrust in health systems and the COVID-19 vaccine (3.9%) and the public did not perceive COVID-19 infection (3.3%). The least number of respondents was less than 1% in which the respondents did not recognize the importance of vaccines (0.9%), believed in homeopathy (0.6%), and could not trust pharmaceutical companies (0.6%).

**Table 3:** Reasons to hesitate about COVID-19 vaccination

Reasons to hesitate about COVID-19 vaccination	Frequency (n)	Percentage (%)
Authorities promote the COVID-19 vaccine for political gain and financial gain (not for people’s health)	8	2.4
Belief in homeopathy	2	0.6
Cannot trust pharmaceutical companies	2	0.6
COVID-19 is a new disease and vaccines have not been tested thoroughly	103	30.9
COVID-19 is not a real disease	8	2.4
Did not perceive COVID-19 infection	11	3.3
Fear of adverse effects	162	48.6
Fear of needle	8	2.4
Mistrust in the health system and COVID-19 vaccine	13	3.9
Natural immunity lasts longer than vaccination	8	2.4
Too young or old for the COVID-19 vaccine	5	1.5
Vaccination is not important	3	0.9

On the other hand, the most common reasons for getting vaccinated were awareness about vaccines (23.1%), followed by a belief that vaccines can stop severe COVID-19 disease (16.8%), and self-protection (14.7%). The least common reason was being vaccinated was belief in pharmaceutical companies (0.6%) (Table 4).

**Table 4:** Reasons to get vaccinated against COVID-19

Reasons to get vaccinated against COVID-19	Frequency (n)	Percentage (%)
Advice from someone	36	10.8
Awareness about vaccines	77	23.1
Become infected by COVID-19	17	5.1
Being forced to take vaccine	37	11.1
Belief in pharmaceutical companies	2	0.6
Free of cost	14	4.2
I believe that I can rely on vaccines to stop severe COVID-19 disease	56	16.8
Religious reasons	4	1.2
Self-protection	49	14.7
Social norms	14	4.2
Trust in the effectiveness of the COVID-19 vaccine	27	8.1

## Discussion

People’s reluctance towards COVID-19 vaccination may cause several problems. Vaccine hesitancy may threaten communities to develop herd immunity [20]. Besides that, COVID-19 vaccine hesitancy may lead to higher mortality in the future. A study by Mesa et al showed that countries with high vaccine hesitancy were predicted to have 7.6 times higher mortality within a two-year period [21].

According to our study, 67.9% of respondents agree that vaccines can control the pandemic. The most common reasons for people getting vaccinated are awareness about vaccines. In addition, many respondents also had a belief that vaccines can stop the occurrence of severe COVID-19 diseases and protect themselves. It can be concluded that the knowledge about vaccines and their effectiveness in protecting against and preventing severe COVID-19 are important aspects in determining whether or not Pakistanis want to get vaccinated. However, there are some challenges in ensuring the same knowledge about COVID-19 vaccines. Lack of education remains the cause of public health problems in developing countries [22]. A better understanding of COVID-19 vaccines increased vaccine acceptance among healthcare workers [23]. Moreover, the spread of false information and conspiracy regarding COVID-19 vaccines was prevalent and widely documented. Technology advancements and social media create opportunities to keep people safe, informed, and connected. However, the same tools also enable and amplify the current infodemic that continues to undermine the global response and jeopardizes measures to control the pandemic. In the digital era, social media is a powerful tool that allows people to share information, including misinformation [4]. Population which lacks digital literacy, making them vulnerable to various types of online and digital propaganda. A previous study showed that Twitter had the highest number of misinformation compared to YouTube and Facebook [24]. This condition is worsened as people re-share the information without prior verification. It can be deduced that Pakistan is fighting two forces: a pandemic and an ‘infodemic’ during the pandemic situation.

Almost half of the respondents rejected COVID-19 vaccines due to their fear of the vaccines’ adverse effects. Side effects of vaccines determine vaccine acceptance, along with infection risk, disease severity, vaccination history, and ethnicity [25]. Similarly, a previous study demonstrated that 58% of unvaccinated respondents in the United States worried about the side effects of COVID-19 vaccines, especially regarding reproductive health and fertility [26]. In European countries, the reported COVID-19 side effects, such as blood clots, caused the suspension of COVID-19 vaccinations [27]. One-third of respondents also thought that COVID-19 vaccines have not been tested thoroughly. Similarly, the previous study demonstrated that vaccine side effects, vaccine safety, and vaccine effectiveness were the concern of people [28-30]. Interestingly, the perception of pharmaceutical companies’ involvement in determining vaccine acceptance and vaccine hesitancy was the least.

The limited number of respondents becomes the limitation of this study. A total of 367 respondents cannot represent the general population of Pakistan. Further study should involve more respondents for better representation of vaccine acceptance or hesitancy among Pakistanis. We also did not assess the location where the respondents came from in Pakistan as the level of education may vary across the geographical location in Pakistan.

## Conclusions

Most Pakistanis accepted the COVID-19 vaccines with the majority contributing by awareness and a belief regarding the protective effects of vaccines. However, the fear against vaccines was also surging along with the spread of misinformation. Even though vaccine hesitancy was still present due to public concern about vaccines' side effects and testing, most of the population showed a belief that the vaccines can stop severe COVID-19 disease. The basic challenge faced by the policymakers of developing countries is the way to utilize limited resources to achieve interconnected goals for managing health recovery, economic crises, and creating environmental sustainability. A well-organized framework, strategic thinking, and planning are required to control COVID-19 challenges in low and middle-income countries.

## References

[1] Rasheed A, Usman T, Niaz S (2021). A review on severe acute respiratory syndrome 2 (SARS CoV-2). Pakistan J Zool.

[2] Hussain Y, Muhammad K, Umer MF (2021). Coronavirus disease 2019 in 5 neighboring limited-resource countries: a financial and health threat. Value Health Reg Issues.

[3] Khan Z, Karataş Y, Rahman H, Qayum M, Alzahrani KJ, Kashif SM (2022). COVID-19 treatments and associated adverse reactions: the need for effective strategies to strengthen pharmacovigilance system in lower- and middle-income countries. Le Pharmacien Hospitalier Clinicien.

[4] Ali I, Shah SA, Siddiqui N (2022). Pakistan confirms first two cases of coronavirus, govt says ‘no need to panic’. https://www.dawn.com/news/1536792.

[5] Khattak S, Khan M, Usman T (2021). Assessment of general populations knowledge, attitude, and perceptions toward the coronavirus disease (COVID-19): a cross-sectional study from Pakistan. Front Med (Lausanne).

[6] Cucinotta D, Vanelli M (2020). WHO declares COVID-19 a pandemic. Acta Biomed.

[7] Rodrigues CM, Plotkin SA (2020). Impact of vaccines; health, economic and social perspectives. Front Microbiol.

[8] El-Elimat T, AbuAlSamen MM, Almomani BA (2021). Acceptance and attitudes toward COVID-19 vaccines: a cross sectional study from Jordan. PLoS ONE.

[9] Andre FE, Booy R, Bock HL (2008). Vaccination greatly reduces disease, disability, death and inequity worldwide. SciELO.

[10] Lopalco PL (2012). Assessing vaccines and vaccination programmes in the field. Italian J Public Health.

[11] MacDonald NE (2015). Vaccine hesitancy: definition, scope and determinants. Vaccine.

[12] Mangrio NK, Alam MM, Shaikh BT (2008). Is Expanded Programme on Immunization doing enough? Viewpoint of health workers and managers in Sindh, Pakistan. J Pak Med Assoc.

[13] Greenfield C (2022). Pakistan launches COVID-19 vaccine campaign. https://www.reuters.com/article/health-coronavirus-pakistan-vaccine-idUSKBN2A225A.

[14] Gul A (2021). Pakistan Starts COVID-19 Inoculation Drive. https://www.voanews.com/a/covid-19-pandemic_pakistan-starts-covid-19-inoculation-drive/6201529.html.

[15] Muhammad SZ, Shaikh N, Asad D (2022). Challenges to mass immunization against COVID-19 in Pakistan: a lower-middle income vaccine-hesitant country. J Glob Health.

[16] VIPER Group (2022). COVID19 Vaccine Tracker Team.

[17] Ali WW, Malik A, Basray R (2021). Assessment of COVID-19 linked fear perception in the community of Pakistan, 1 June to 31 July. Global Biosecurity.

[18] Raosoft Raosoft (2004). Sample size calculator. http://www.raosoft.com/samplesize.html.

[19] Worldometers Worldometers (2022). Pakistan population. https://www.worldometers.info/world-population/pakistan-population/.

[20] Gerretsen P, Kim J, Quilty L (2021). Vaccine hesitancy is a barrier to achieving equitable herd immunity among racial minorities. Front Med (Lausanne).

[21] Mesa DO, Hogan AB, Watson OJ (2022). Modelling the impact of vaccine hesitancy in prolonging the need for non-pharmaceutical interventions to control the COVID-19 pandemic. Commun Med.

[22] Petrakova A, Sadana R (2007). Problems and progress in public health education. Bull World Health Organ.

[23] Malik A, Malik J, Ishaq U (2021). Acceptance of COVID-19 vaccine in Pakistan among health care workers. PLoS One.

[24] Brennen Brennen, Scott AJ, Simon FM (2020). Types, sources, and claims of COVID-19 misinformation. Oxford. Oxford University Press.

[25] Nguyen T, Henningsen KH, Brehaut JC, Hoe E, Wilson K (2011). Acceptance of a pandemic influenza vaccine: a systematic review of surveys of the general public. Infect Drug Resist.

[26] Diaz P, Zizzo J, Balaji NC, Reddy R, Khodamoradi K, Ory J, Ramasamy R (2022). Fear about adverse effect on fertility is a major cause of COVID-19 vaccine hesitancy in the United States. Andrologia.

[27] Wise J (2021). Covid-19: European countries suspend use of Oxford-AstraZeneca vaccine after reports of blood clots. BMJ.

[28] Wagner AL, Huang Z, Ren J (2021). Vaccine hesitancy and concerns about vaccine safety and effectiveness in Shanghai, China. Am J Prev Med.

[29] Yasmin F, Asghar W, Babar MS (2021). Acceptance rates and beliefs toward COVID-19 vaccination among the general population of Pakistan: a cross-sectional survey. Am J Trop Med Hyg.

[30] Ali S, Pasha SA, Khalid A (2022). COVID-19, vaccination, and conspiracies: a micro-level qualitative study in Islamabad, Pakistan. Yale J Biol Med.

